# Clinical Interpretation of Cardiopulmonary Exercise Testing: Current Pitfalls and Limitations

**DOI:** 10.3389/fphys.2021.552000

**Published:** 2021-03-18

**Authors:** J. Alberto Neder, Devin B. Phillips, Mathieu Marillier, Anne-Catherine Bernard, Danilo C. Berton, Denis E. O’Donnell

**Affiliations:** ^1^Laboratory of Clinical Exercise Physiology and Respiratory Investigation Unit, Queen’s University and Kingston General Hospital, Kingston, ON, Canada; ^2^Division of Respirology, Federal University of Rio Grande do Sul, Porto Alegre, Brazil

**Keywords:** exercise, dyspnea, lung function, cardiopulmonal capacity, exercise test interpretation

## Abstract

Several shortcomings on cardiopulmonary exercise testing (CPET) interpretation have shed a negative light on the test as a clinically useful tool. For instance, the reader should recognize patterns of dysfunction based on clusters of variables rather than relying on rigid interpretative algorithms. Correct display of key graphical data is of foremost relevance: prolixity and redundancy should be avoided. Submaximal dyspnea ratings should be plotted as a function of work rate (WR) and ventilatory demand. Increased work of breathing and/or obesity may normalize peak oxygen uptake (V̇O_2_) despite a low peak WR. Among the determinants of V̇O_2_, only heart rate is measured during non-invasive CPET. It follows that in the absence of findings suggestive of severe impairment in O_2_ delivery, the boundaries between inactivity and early cardiovascular disease are blurred in individual subjects. A preserved breathing reserve should not be viewed as evidence that “the lungs” are not limiting the subject. In this context, measurements of dynamic inspiratory capacity are key to uncover abnormalities germane to exertional dyspnea. A low end-tidal partial pressure for carbon dioxide may indicate either increased “wasted” ventilation or alveolar hyperventilation; thus, direct measurements of arterial (or arterialized) PO_2_ might be warranted. Differentiating a chaotic breathing pattern from the normal breath-by-breath noise might be complex if the plotted data are not adequately smoothed. A sober recognition of these limitations, associated with an interpretation report free from technicalities and convoluted terminology, is crucial to enhance the credibility of CPET in the eyes of the practicing physician.

## Introduction

Cardiopulmonary exercise testing (CPET) might be helpful in uncovering the causes of exercise intolerance in patients with (or at risk of) cardiorespiratory diseases (Marciniuk et al., 2013). In the pulmonology practice, CPET is usually requested as part of the work-up for unexplained or “out-of-proportion” dyspnea (Neder et al., 2019a). The test, however, rarely pinpoints to a specific diagnosis; thus, it should be better considered as part of the initial assessment to guide further investigative efforts (if required). Unfortunately, however, CPET remains poorly understood and, therefore, largely underused in clinical practice. Apart from operational issues (e.g., high costs, limited availability, and poor reimbursement relative to time spent on the test), there are several shortcomings on testing interpretation which have not helped to improve this state of affairs (Neder et al., 2018b). In this concise *Perspective*, some of these pitfalls and challenges are outlined: owing to the absence of large randomized trials exploring CPET limitations, they were selected based on our long-standing experience with CPET reading and teaching. Moreover, we focused on the limitations more likely to impact on clinical-decision making (Table 1). Of note, we specifically assume that the reader is already familiar with clinical CPET interpretation; thus, a thorough discussion of interpretative strategies is beyond the scope of this viewpoint article. When feasible, we provide a brief account of the available strategies to avoid key interpretative mistakes (Table 1). If the limitations are deemed insurmountable at this point in time, we caution the reader that the best (and more honest) approach is to refrain from testing over-interpretation. Finally, representative examples are not provided due to space constraints.

**Table 1:** Selected pitfalls and limitations on cardiopulmonary exercise testing (CPET) interpretation which are more likely to negatively impact on clinical-decision making in respiratory medicine.

Pitfall/limitation	Potential consequence	Recommended approach
Over-Reliance on Rigid Interpretative Algorithms	Misdiagnosis of the mechanisms leading to exercise intolerance	Identify cluster of findings indicative of syndromic patterns of dysfunction
Incorrect Display of Graphical Data	Distraction and redundancy	Focus on the dynamic relationships more likely to expose the patterns of dysfunction
Considering Dyspnea a Secondary Outcome	Poor diagnostic yield in patients under investigation for indetermined dyspnea	Obtain submaximal dyspnea scores which should be expressed as a function of both work rate and ventilation
Misinterpretation of a “Preserved” Peak V̇O_2_ as Evidence of Normality	False negative for exercise intolerance	Carefully review all available data, even in the presence of a “preserved” peak V̇O_2_
Ignoring the Effects of Obesity on the ∆V̇O_2_/∆ Work Rate Relationship	As above	As above; value potential decrements in peak work rate
Failure to Recognize the Poor Diagnostic Performance of CPET in Indicating Cardiac Disease	Misdiagnosis of potential cardiovascular abnormalities	A cautious, non-committal approach when ruling in or out a cardiac disease when the pre-test likelihood of disease is unclear
Misdiagnosis of Mechanical-Ventilatory Limitation	Failure to recognize an etiologic role for “the lungs” in limiting the subject	Routine measurement of dynamic IC (operating lung volumes)
Under-Recognition of the Limitations of Non-invasive Assessment of Pulmonary Gas Exchange	Over- or under-calling of gas exchange inefficiency for O_2_ or CO_2_	Recognize that measurements of arterial (or arterialized) blood gases might be warranted
Over- or Under Recognition of Chaotic Breathing Pattern/Dysfunctional Breathing	Misdiagnosis of behavioral/psychogenic abnormalities	Adequate data smoothing; apply a gestalt approach to breathing pattern analysis

V̇O_2_, oxygen uptake; IC, inspiratory capacity.

## Discussion

### Over-Reliance on Rigid Interpretative Algorithms

There is a pervasive sense that, in similarity with pulmonary function tests, CPET can be meaningfully interpreted considering some dichotomous decision nodes in a hierarchic evaluation tree. In fact, if any, the clinical interpretation of CPET is full of *chance nodes*: from these nodes, one can only infer the meaning of a certain result given a set of pre-existing conditions. In other words, no variable holds discriminative properties when seen in isolation, i.e., without a proper estimation of the pre-test likelihood of abnormality (Bayes theorem). More realistically, cluster of findings may indicate the presence of certain patterns: (a) a normal maximal or sub-maximal test, (b) obesity, (c) O_2_ delivery/utilization impairment, (d) mechanical-ventilatory impairment, (e) pulmonary gas exchange impairment, and (f) dysfunctional breathing-hyperventilation disorder (Neder et al., 2018a). The referring physician should be specifically aware that individual features overlap across diseases. It is the referring physician’s responsibility to amalgamate the described pattern(s) of abnormalities on his/her diagnostic plan or prognostic assessment. Although these recommendations seem rather obvious, they are not easily implemented in practice either because: (a) the requester over-estimates the test sensitivity/specificity and/or (b) the reader fails to recognize its important limitations. A detailed account of testing interpretation in light of these precautionary considerations is provided by Neder et al. (2018a).

### Incorrect Display of Graphical Data

The great majority of CPETs performed by respiratory patients are symptom-limited. Thus, appreciation of the sensory responses to exercise is an integral part of testing interpretation (O’Donnell et al., 2019). The fundamental task of the reader is to select the more appropriated dependent variables in response to their physiological determinants (or, at least, their closest correlates) taken into consideration the principles of biological plausibility and simplicity (Occam’s razor principle). In order to maximize the yield of information and minimize distraction, redundancy must be avoided. Although rapidly-incremental tests (frequently following a ramp forcing regimen; Laveneziana et al., 2017) are almost universally used nowadays, in practice, there is an ample variability in the rates of work increment and stages duration across laboratories. Thus, work rate (WR) and time might not always be the best independent variables to judge the response normalcy in individual subjects. The test is based on the fundamental physiological principle that the heart and the lungs ultimately support the uptake of O_2_ and release of CO_2_ – which vary markedly for a given work rate or testing time elapsed. Thus, it could be argued that metabolic-cardiovascular responses, minute ventilation (V̇E), and lung mechanics/breathing pattern in the *y*-axis are better expressed relative to their closest determinants in the *x*-axis, i.e., oxygen uptake (V̇O_2_; Whipp and Ward, 1982), carbon dioxide output (V̇CO_2_; Whipp, 1977), and V̇E (O’Donnell et al., 2017), respectively.

**Figure 1:**
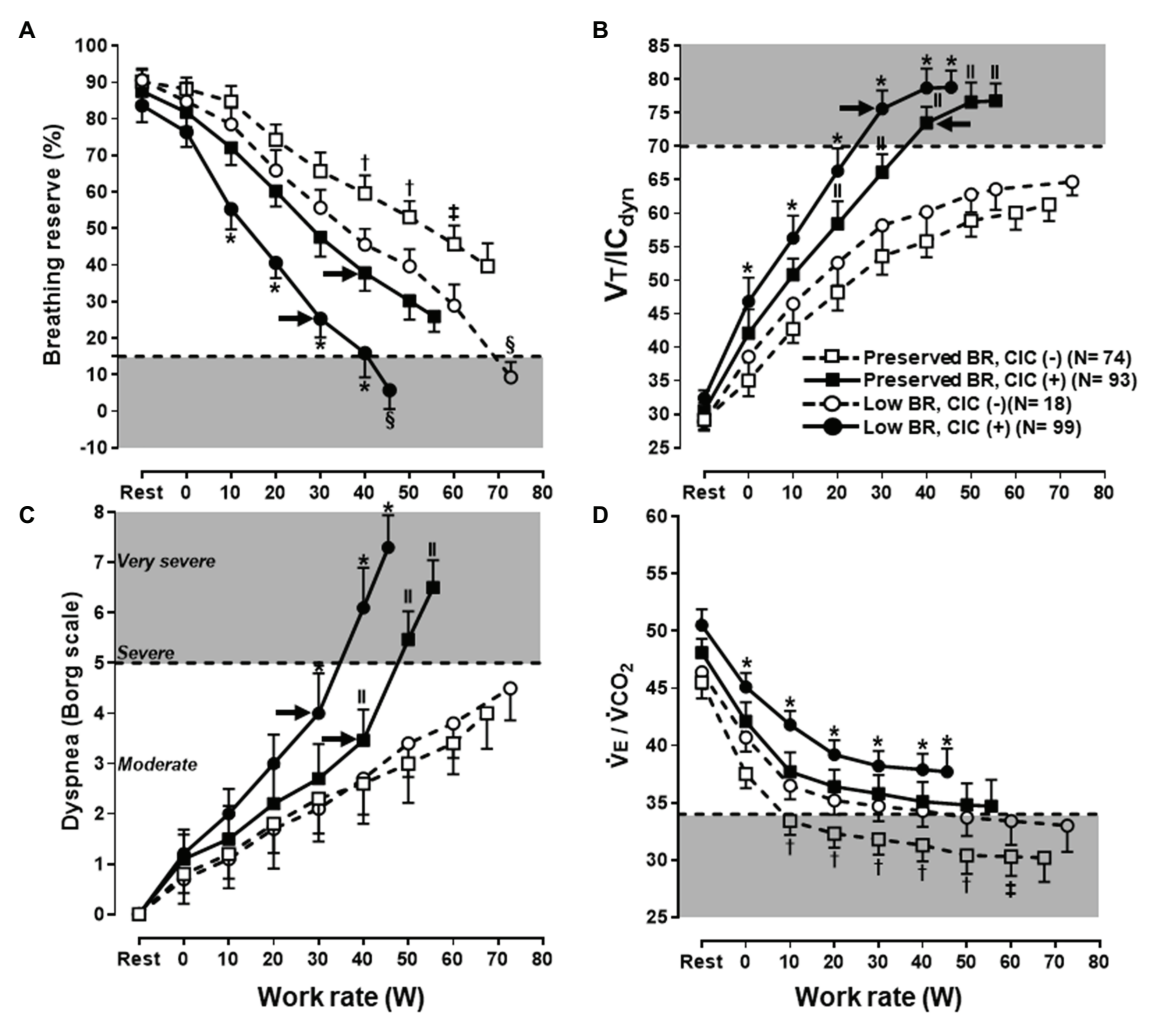
Selected ventilatory and sensory responses to symptom limited incremental CPET in subjects under investigation for exertional dyspnea. Subjects were separated according to the combination of preserved or low peak breathing reserve (BR) vs. absence (−) or presence (+) of critical inspiratory constraints (CIC). Note that a low BR **(A)** was found in subjects who either low or high levels of dyspnea **(C)**; conversely, a sizeable fraction of subjects with preserved BR reported severe dyspnea. Regardless of the BR, subjects who develop CIC **(B)** and/or presented with poor ventilatory efficiency [high ventilation (V̇E)/carbon dioxide output (V̇O_2_) in **D**] were consistently more dyspneic. Note the additive effects of these physiological abnormalities. Shaded areas represent the limits for a low BR, CIC, high dyspnea burden, and poor ventilatory efficiency, respectively. The arrows in **(A–C)** indicate the exercise intensities associated with an upward inflection in dyspnea ratings in CIC(+) subjects. See the text for further elaboration. Data are mean ± SEM. ^*^p < 0.05 vs. all groups; † vs. low BR, CIC(−) and preserved BR, CIC(+); ‡ vs. low BR, CIC(−); § vs. preserved BR, CIC(−) and preserved BR, CIC(+); ll vs. low BR, CIC(−) and preserved BR, CIC(−). VT, tidal volume; IC, inspiratory capacity. Reproduced, with the permission of the publisher, from Neder (2019a).

The Wasserman et al. (1987) nine-panels remain the most popular display. It has, however, important limitations which are frequently overlooked (Dumitrescu and Rosenkranz, 2017):

- the panels are heavily biased to depict metabolic/cardiovascular responses: not less than five graphs are basically devoted to the identification of gas exchange and ventilatory thresholds (Wasserman et al., 1973; Beaver et al., 1986);
- V̇O_2_ and work rate are both expressed as a function of time. Thus, the fundamental relationship of clinical interest [V̇O_2_ (*y*) vs. work rate (*x*); Whipp and Ward, 1982] is not shown. Even if V̇O_2_-to-work rate ratio is correctly scaled to 10:1, significant departures from linearity in V̇O_2_ (i.e., lack of parallel increase in V̇O_2_ as related to work rate) might not be readily apparent in patients with poor exercise tolerance;
- tidal volume (VT) is plotted as a function of V̇E. VT is also compared to vital capacity (VC) and resting inspiratory capacity (IC), whereas maximal voluntary ventilation (MVV) is shown as the upper limit for V̇E. Resting IC, however, is not the correct benchmark to contrast against the VT trajectory as IC usually increases with exercise in healthy subjects or decreases in patients showing expiratory flow limitation (Guenette et al., 2013). VC is substantially greater than IC and the former does not allow a clear recognition of the limits for VT expansion, i.e., exercise IC (O’Donnell et al., 2019). MVV is not a consistent ceiling for a V̇E increase and severe dyspnea might arise in patients with still-preserved breathing reserve (Neder et al., 2019a,b; see also Misdiagnosis of Mechanical-Ventilatory Limitation section; Figure 1); and
- the operating lung volumes and dyspnea readings are ignored (see also Considering Dyspnea a Secondary Outcome section).

Alternative displays which avoid these errors and omissions are provided elsewhere (O’Donnell et al., 2017, 2019).

### Considering Dyspnea a Secondary Outcome

The burden to provide a resolution to complex cases of persistent shortness of breath is frequently directed to the pulmonologist (Mahler and O’Donnell, 2015). CPET is a non-invasive procedure which was meant to uncover the causes of exertional breathlessness (Wasserman and Whipp, 1975; Wasserman et al., 1987; Jones, 1988; Weisman and Zeballos, 1996). Indeed, the test measures a multitude of physiological responses important for the genesis of the symptom; thus, at least theoretically, CPET is well-suited to the task (Arena and Sietsema, 2011). In this context, it is rather axiomatic that a special attention should be given to the measurement and interpretation of dyspnea scores. Unfortunately, this is more an exception than a rule in most clinical laboratories nowadays.

In order to fully recognize the advantages of incorporating dyspnea readings on CPET interpretation, it is instructive to consider some basic neurobiological concepts. At a close inspection, exertional dyspnea boils down to a heightened sense of inspiratory load (Campbell and Howell, 1963; Killian and Campbell, 1983). More specifically, the respiratory controller (i.e., pontine-medullary centers and their cortical-limbic connections) continuously appraise “how much ventilation” is performed at a given point in the time. Such quantitative perspective of the load is influenced by chemo-stimulation of central and peripheral receptors (Plataki et al., 2013) and the efferent motor output to the inspiratory muscles (Killian and Campbell, 1983). In the absence of critical mechanical constraints, increased reflex chemostimulation (Plataki et al., 2013) translates into excessive ventilatory response relative to metabolic demand (Neder et al., 2015; Rocha et al., 2017). Consequently, when the increased drive to breathe can be freely converted into the act of breathing, patients tend to report higher dyspnea for a given work rate *but* similar dyspnea for a given V̇E compared to normal subjects (Kearon et al., 1991; Killian et al., 1992). Conversely, when VT becomes positioned close to the upper reaches of the S-shaped pressure-volume relation of the relaxed respiratory system, compliance decreases, the inspiratory muscles are functionally weakened, and intolerable dyspnea quickly ensues. As a corollary, dynamic mechanical constraints lead to higher dyspnea ratings as a function of *both* work rate and V̇E (O’Donnell et al., 2019; Plachi et al., 2020). Thus, dyspnea should be carefully measured and plotted as a function of exercise intensity as reflected by increases in power output and ventilatory demand. Normative values have been recently published (Neder et al., 2020).

### Misinterpretation of a “Preserved” Peak V̇O_2_ as Evidence of Normality

Peak V̇O_2_ is highly dependent on the averaging method used to decrease the variability of breath-by-breath data. As expected, the shorter the averaging interval (and the lower the number of breaths considered for averaging), the higher the peak V̇O_2_. Unfortunately, there are no consistent recommendations among existing guidelines on the averaging method. In practice, the most common settings range from 10- to 60-s periods: rolling averages of 15–20 s usually provide reproducible estimates of peak V̇O_2_ in respiratory patients. Peak V̇O_2_ is usually interpreted without the help of previous values for a meaningful longitudinal comparison. Thus, substantial loss of aerobic capacity might be missed if an impaired subject had, at an unknown baseline, a supra-normal peak V̇O_2_ (Neder et al., 1998). A peak V̇O_2_ within expected limits may coexist with extensive sub-maximal abnormalities; in fact, some of them (e.g., increased work of breathing) may increase “whole-body” V̇O_2_, bringing the peak value up to the limits of reference (Neder et al., 2018a).

### Ignoring the Effects of Obesity on the ∆V̇O_2_/∆ Work Rate Relationship

Obese subjects expend more O_2_ to perform a given amount of external work as they need to displace a larger mass against gravity (Whipp and Davis, 1984). High V̇O_2_/work rate ratio in an obese subject may result in normal or even increased peak V̇O_2_ (L/min) despite a low peak work rate. This is not easily fixed by attempts to “correct” V̇O_2_ for the mass displaced, i.e., total body and leg mass on treadmill and cycle ergometer, respectively (Neder et al., 2000). Expressing V̇O_2_ as a function of total body mass (ml/min/kg) is a more reasonable approach in treadmill-based tests. Conversely, it tends to penalize the obese exercising on a stationary bike (Neder et al., 1998). A valid alternative is to use reference values for peak V̇O_2_ based on height or ideal body weight and analyze results in percentage predicted (Hansen et al., 1984). In an obese subject showing low peak work rate but preserved peak V̇O_2_, the former is likely to better reveal subject’s functional capacity. This is particularly true if peak work rate is predicted from studies which included a large number of obese subjects (e.g., Koch et al., 2009; Myers et al., 2017).

### Failure to Recognize the Poor Diagnostic Performance of CPET in Indicating Cardiac Disease

The accuracy at which the syndrome of impaired O_2_ delivery/utilization reflects cardiocirculatory disease is based on the assumptions that: (a) the “downstream” (to the heart) consequences of these diseases are always present and (b) isolated or concurrent abnormalities at the muscle level can be differentiated from those occurring “upstream.” Unfortunately, these premises are not consistently met in practice. It should also be recognized that non-invasive CPET only measures one of the three variables that are known to determine V̇O_2_: heart rate (HR), stroke volume, and O_2_ extraction. Deficits in stroke volume can be compensated by increases in HR and/or O_2_ extraction; moreover, HR can be impaired in the absence of structural cardiac disease (see also below). Thus, in the absence of findings suggestive of severe (“out-of-proportion” to physical deconditioning) impairment in O_2_ delivery, the boundaries between inactivity and early cardiovascular disease are blurred in individual subjects. Thus, whereas a CPET deemed highly-suggestive of cardio-circulatory dysfunction deserves further investigation, a normal test in a subject with high pre-test likelihood of disease should be better seen as “not consistent with current moderate-to-severe dysfunction” (Neder et al., 2018a,b).

It should be explicitly recognized that a sizeable fraction of patients referred to clinical CPET have their resting and exertional HR under pharmacological or external control, e.g., β-blockers and pacemakers, respectively. Moreover, the prevalence of chronotropic incompetence has increased markedly in tandem with inactivity, obesity, and metabolic disorders (Brubaker and Kitzman, 2011). Thus, CPET variables based on HR, including the double-product (HR times systolic blood pressure), should be viewed with caution in these patients. Specifically, shallow ΔHR/ΔV̇O_2_ and/or high O_2_ pulse should not be erroneously assumed as indicative of normality. Quite the opposite: a severely blunted ΔHR/ΔV̇O_2_ in a CPET interrupted with objective and subjective evidences of maximal effort should be clinically valued as a potential source of exercise intolerance (Brubaker and Kitzman, 2011).

### Misdiagnosis of Mechanical-Ventilatory Limitation

Maximal voluntary ventilation is a poor index of maximum breathing capacity during exercise (Babb and Rodarte, 1993). Regardless of the clinical scenario, relying on single cut-off of V̇E/MVV ratio to rule out ventilatory limitation might be misleading. The ratio correlates poorly to exertional dyspnea in individual patients with both obstructive and restrictive disorders (O’Donnell et al., 2014; Faisal et al., 2016): as discussed above, it might serve as a metric of the quantitative (“*how much V̇E*”) mechanisms of dyspnea, but it is insensitive to its qualitative foundations (“*how appropriate V̇E is performed*”). Some dyspnoeic patients with chronic obstructive pulmonary disease (COPD), particularly those with mild-moderate airflow limitation (O’Donnell et al., 2016), stop exercising with preserved V̇E/MVV but with clear evidences of constrained mechanics (Figure 1; O’Donnell et al., 2017; Neder et al., 2019b) Moreover, a still-preserved end-exercise V̇E/MVV might be relevant for dyspnea and exercise intolerance if reached at a low peak work rate Thus, a high V̇E/MVV might be valued to indicate low ventilatory reserves but a low V̇E/MVV should never be considered as the definitive proof that mechanical-ventilatory abnormalities are not relevant to patient’s dyspnoea (Neder et al., 2019a).

The assessment of the operating lung volumes is based on the basic premise that a full inspiratory effort has been performed (Guenette et al., 2013). For instance, progressively lower IC due to inspiratory muscle weakness may be misinterpreted as indicative of dynamic hyperinflation. A common mistake is the belief that lack of IC decrement from rest signal for a normal response. Due to pronounced gas trapping at rest, however, patients with advanced COPD may not be able to further decrease IC during exercise (O’Donnell et al., 2017). A plateau in VT at higher operating lung volumes should raise the suspicion of the attainment of critical inspiratory constraints (Casaburi and Rennard, 2015); nevertheless, VT also tends to plateau after the respiratory compensation point (Neder and Stein, 2006). Thus, it is advisable to double-check whether such a plateau coincides or not with near-maximum end-inspiratory volumes, e.g., VT/exercise IC ≥ 0.8 and/or end-inspiratory lung volume/TLC ≥ 0.9 (Guenette et al., 2013). The maximum flow-volume loop provides a poor frame of reference of the flow reserves at a given lung volume on effort, particularly in the presence of moderate to severe airflow limitation (Johnson et al., 1999). In practice, it is commonly valuable to assess changes on tidal expiratory limb’s morphology (from convex to rectified or concave; Varga et al., 2016) and eventual leftward shifts in the tidal flow-volume loop as exercise progresses, i.e., dynamic hyperinflation.

### Under-Recognition of the Limitations of Non-invasive Assessment of Pulmonary Gas Exchange

Due to the sigmoid shape of the O_2_ dissociation curve and the high noise-to-signal ratio of pulse oximeters on exertion, mild-moderate decrements in the arterial partial pressure for O_2_ (PaO_2_) might be missed by measurements of the arterial O_2_ saturation by this method (SpO_2_). A pattern of impaired O_2_ delivery/utilization might be seen in “respiratory” patients with severe exertional hypoxemia. The end-tidal partial pressure for CO_2_ (PETCO_2_) is a particularly poor indicator of PaCO_2_ in patients with respiratory diseases (ERS Task Force et al., 2007). Thus, low PETCO_2_ values may indicate high ventilation/perfusion or alveolar hyperventilation, i.e., dissimilar phenomena with opposite clinical implications. Conversely, a high PETCO_2_ might either reflect the late emptying of poorly-ventilated units with higher alveolar PCO_2_ or alveolar hypoventilation (Hansen et al., 2007). In addition, a superficial and fast breathing pattern may decrease PETCO_2_ since less alveolar air is sampled and the expiratory time becomes too short, i.e., there is not enough time for PCO_2_ to raise up to its highest value (Whipp and Ward, 1982). This explains why “automatic,” non-invasive dead space/VT ratio (using PETCO_2_) underestimates the true dead space/VT in patients with ventilation-perfusion inequalities (Lewis et al., 1994), i.e., a “preserved” non-invasive dead space/VT is not useful to rule out poor gas exchange efficiency. Minimally-invasive or non-invasive alternatives to PaCO_2_ include capillary (arterialized) PCO_2_ (McLoughlin et al., 1992) or transcutaneous PCO_2_ (Hoffmann et al., 1990). If these techniques are used, serial measurements (at least three) are particularly useful to track the trajectory during incremental exercise (Rocha et al., 2017).

### Over- or Under Recognition of Chaotic Breathing Pattern/Dysfunctional Breathing

There is a large heterogeneity on the presence and extension of dysfunctional breathing and hyperventilation across individuals, including the timing as related to rest and/or exercise. Detailed normative values for the timing and pattern of breathing at a given V̇E have long been published (Neder et al., 2003); unfortunately, they are not commonly available in commercial software. As expected, differentiating a chaotic breathing pattern from the normal breath-by-breath noise might be complex if the plotted data is not adequately smoothed. Unless potential underlying abnormalities have been carefully excluded (e.g., neuromuscular disease, respiratory muscle weakness, asthma, and paradoxical vocal cord motion; Boulding et al., 2016), it is prudent to avoid labeling the phenomenon as “primary” or “merely psychogenic.” Specific care should be taken to rule out a cyclic pattern of V̇E oscillation which represents an important sign of cardiovascular disease and/or breathing control instability (periodic breathing; Leite et al., 2003) Relative to the later phenomenon, care should be taken to depict plot the V̇E axis (here, as an exception, vs. work rate or time) with sufficient resolution to appreciate the cycling changes. Owing to the fact that the ventilatoty and metabolic data oscillate in phase (Neder et al., 2003), period breathing is usually missed if only V̇E vs. V̇CO_2_ is examined.

## Conclusion

There remains a long way for CPET to be widely recognized as a clinically-useful tool for the investigation of the mechanisms of dyspnea and exercise intolerance in patients with chronic cardiorespiratory disease. As discussed in this *Perspective*, we have advanced substantially in the identification of common pitfalls for testing interpretation. We are also more aware of CPET limitations, particularly in this era of advanced imaging and invasive diagnostic procedures. A sober recognition of these limitations, associated with a final report free from technicalities and convoluted terminology, is crucial to enhance the credibility of CPET in the eyes of the practicing physician.

## Data Availability Statement

The original contributions presented in the study are included in the article/supplementary material, further inquiries can be directed to the corresponding author.

## Author Contributions

JAN wrote the first draft. All authors were involved in the manuscript preparation, contributed to the article, and approved the submitted version.

### Conflict of Interest

The authors declare that the research was conducted in the absence of any commercial or financial relationships that could be construed as a potential conflict of interest.
